# 

**Published:** 1881-05

**Authors:** 


### Table of Contents,

ORIGINAL COMMUNICATIONS.

REFLEX NERVOUS DISTURBANCES. By Robt. W. Westmoreland, M.D., Atlanta, Ga.......... 65

REPORTS OF SOCIETIES.

Philadelphia County Medical Society.......... 68

Philadelphia Academy ot Surgery.......... 72

New York Academy of Medicine.......... 76

Medico-Chirurgical Society of Louisville, Ky.......... 85

West Chicago Medical Society.......... 89

SELECTIONS.

Mechanical Atresia Vaginæ.......... 91

The Import of the Sweating of Consumptives.......... 93

The Heatonian Method for the Radical Cure of Hernia.......... 94

Prevention of Diphtheria.......... 101

Precautions in Dispensing Poisons, etc.......... 107

The Hygiene of Old Age, with Special References to Prostatic Hypertrophy and Secondary Disease of the Bladder.......... 111

Cancer of the Cervix Uteri.......... 117

EDITORIAL.

The Relations Between Druggists and Physicians.......... 127

